# 1155. Coadministration of Intranasal M2SR (M2-deficient Single Replication) Investigational Influenza Vaccine with Fluzone High Dose Induces Superior Immune Responses to Fluzone High Dose Alone in 65-85 Year Old Adults

**DOI:** 10.1093/ofid/ofad500.995

**Published:** 2023-11-27

**Authors:** Joseph Eiden, Carlos Fierro, Alexander White, Matthew Davis, Margaret Rhee, Mark Turner, Bryan Murray, Renee Herber, Roger Aitchison, David Marshall, Michael Moser, Pamuk Bilsel

**Affiliations:** FluGen, Inc., Madison, Wisconsin; Johnson County Clin-Trials, Lenexa, Kansas; Progressive Medical Research, Port Orange, Florida; Rochester Clinical Research, Rochester, New York; Velocity Clinical Research, Cleveland, Ohio; Velocity Clinical Research, Cleveland, Ohio; Boyds Consultants, Crewe, England, United Kingdom; FluGen, Inc., Madison, Wisconsin; North Rim Consulting, Longmont, Colorado; FluGen, Inc., Madison, Wisconsin; FluGen, Inc., Madison, Wisconsin; FluGen, Inc., Madison, Wisconsin

## Abstract

**Background:**

Adults over age 65 years exhibit increased susceptibility to influenza viruses, accounting for 70-85% of annual influenza-related US fatalities. Licensed vaccines mainly induce serum humoral immunity and remain the primary method of influenza prevention, but vaccine effectiveness (VE) remains particularly low in older individuals (**Fig 1**). Stimulation of mucosal antibodies and T cells, immune effectors also associated with protection against influenza, are anticipated to enhance VE.

**Figure:**
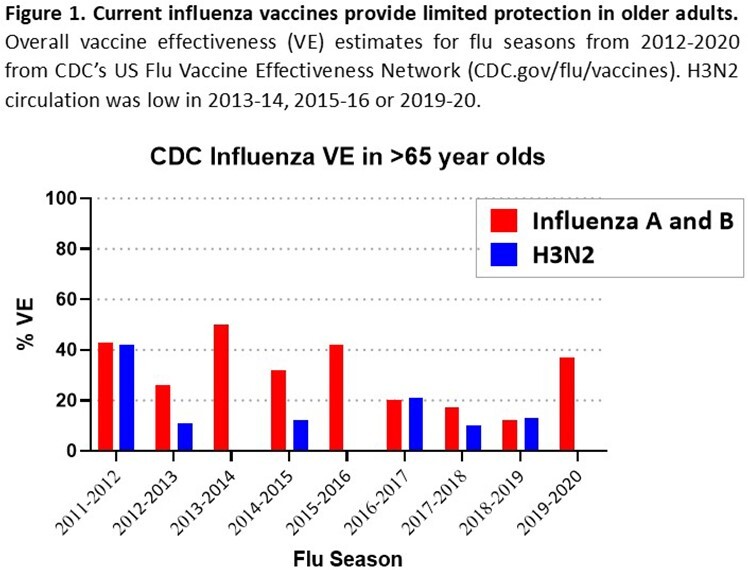

**Methods:**

A randomized, double-blind, double-dummy, placebo-controlled Phase 1b study (NCT05163847) was conducted at 6 US study sites with the investigational intranasal M2SR (M2 deficient Single Replication) and Fluzone High Dose (HD) vaccine that contained HA & NA from A/Cambodia/e0826360/2020 (Cam2020). Adults aged 65-85 years (n = 305) received either one administration of Cam2020 M2SR alone, Cam2020 M2SR along with HD, HD alone, or placebo (**Table 1**). Demographics were comparable across cohorts (**Table 2)**.

**Figure:**
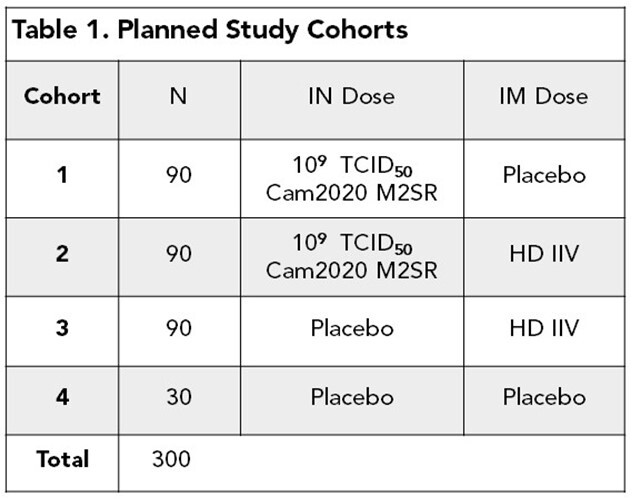

**Figure:**
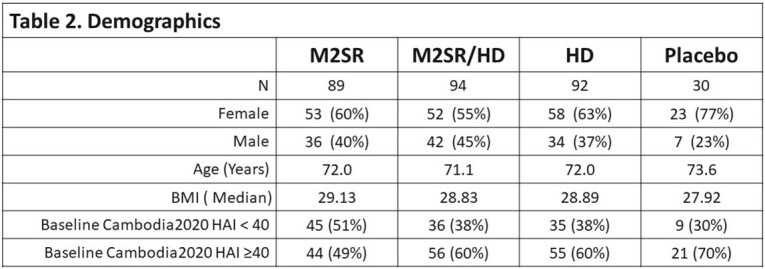

**Results:**

Study vaccination was well-tolerated. Intranasal M2SR generated significantly increased serum HAI and NAI antibodies compared with placebo (**Fig 2**). Moreover, M2SR coadministered with HD induced HAI and NAI antibodies in significantly higher proportion of subjects than HD alone (**Fig 2**). M2SR alone and M2SR coadministered with HD elicited significant mucosal secretory IgA (sIgA) antibodies against vaccine antigen, Cam2020, and drifted H3N2 Darwin2021 strain (**Fig 3**). Similarly, T cells positive for IFNg and GrzB secretion were significantly induced by M2SR only or by M2SR coadministered with HD (**Fig 4**). HD alone did not induce mucosal antibodies or T cell responses (**Figs 3 & 4**).

**Figure:**
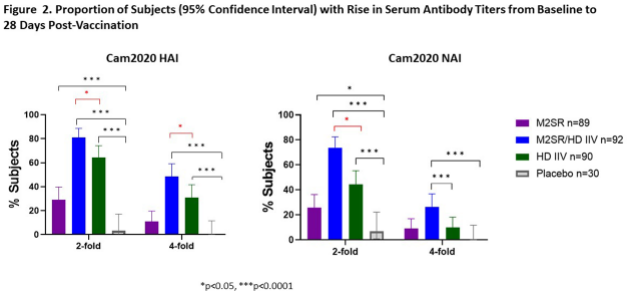

**Figure:**
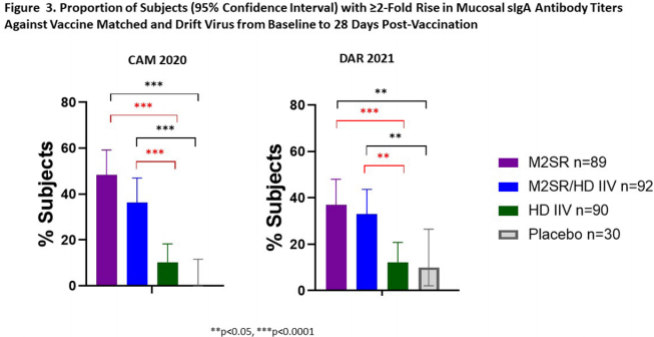

**Figure:**
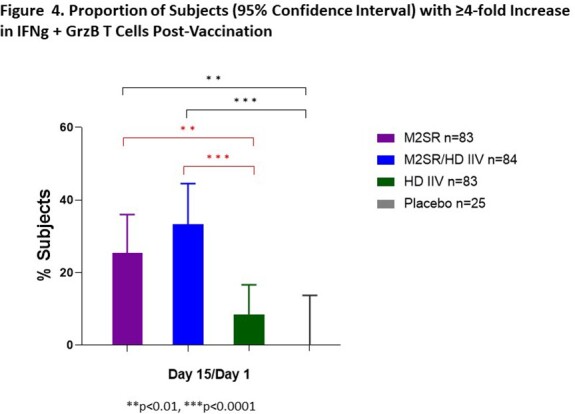

**Conclusion:**

Coadministration of intranasal M2SR with intramuscular Fluzone HD resulted in a multi-faceted immune response in older adults aged 65-85 years old with a higher proportion of responders across all immune measurements compared with either vaccine alone. Coadministration of the two vaccines resulted in significant rises in serum HAI and NAI titers compared to HD alone (**Fig 5**). These broad immune responses support the potential for the safe coadministration of M2SR with HD to enhance protection against influenza infection in this highly vulnerable age group.

**Figure:**
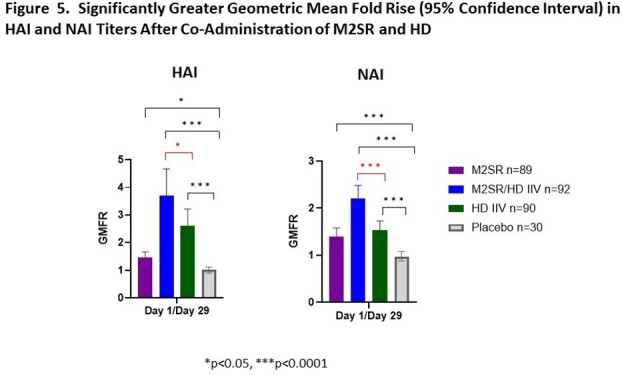

**Disclosures:**

**Joseph Eiden, MD, PhD**, FluGen, Inc.: Advisor/Consultant **Bryan Murray, M.D.**, FluGen, Inc.: Advisor/Consultant **Renee Herber, B.S.**, FluGen, Inc.: Salary **Roger Aitchison, ScM**, FluGen, Inc.: Advisor/Consultant **David Marshall, B.S.**, FluGen, Inc.: salary **Michael Moser, Ph.D.**, FluGen, Inc.: salary **Pamuk Bilsel, Ph.D.**, FluGen, Inc.: salary

